# Significance of DNMT3b in Oral Cancer

**DOI:** 10.1371/journal.pone.0089956

**Published:** 2014-03-13

**Authors:** Wen-Cheng Chen, Miao-Fen Chen, Paul-Yang Lin

**Affiliations:** 1 Department of Radiation Oncology, Chang Gung Memorial Hospital, Chiayi, Taiwan; 2 Chang Gung University, College of medicine, Taoyuan, Taiwan; 3 Department of Pathology, Chang Gung Memorial Hospital, Chiayi, Taiwan; Sapporo Medical University, Japan

## Abstract

The aim of this study was to explore specific molecular markers that could lead to new insights into the identification of innovative treatments. The role of DNMT3b and its predictive power in the prognosis of oral cancer were identified. Human oral cancer cell lines including SCC4 and SCC25 were selected for cellular experiments. Changes in tumor growth, aggressiveness and the responsible signaling pathway were investigated *in vitro* and *in vivo*. Furthermore, 125 oral cancer tissue specimens were analyzed using immunohistochemical staining on tissue microarray slides, and correlations calculated between the level of DNMT3b and the clinical outcome of patients. Our data revealed that inhibition of DNMT3b resulted in slower tumor growth, attenuated tumor invasion ability and epithelial mesenchymal transition, as determined by *in vitro* and *in vivo* experiments. Activated IL-6 signaling might be responsible to the induction of DNMT3b overexpression on oral cancer. Regarding clinical data, the incidence of DNMT3b immunoreactivity in oral cancer specimens was significantly higher than in non-malignant epithelium, and positively linked to expression of IL-6. Furthermore, expression of DNMT3b was significantly linked with the risk of lymph node involvement, disease recurrence and shorter survival in patients with pathological stage III-IV oral cancer. In conclusion, IL-6 –DNMT3b axis could be used to predict the prognosis of oral cancer in clinics, and targeting DNMT3b could represent a promising treatment strategy.

## Introduction

The most frequently oral cancers are oral squamous cell carcinomas (OSCC) which are the most malignant tumors of the Head and Neck. This aggressive epithelial neoplasm is associated with severe morbidity. Post-operative chemoradiotherapy (CCRT) results in better loco-regional control and survival for locally advanced head and neck cancer patients with high-risk factors [1]. Despite considerable advances in treatment, 40–50% of patients with locally-advanced disease relapse with local or distant disease progression [2]. Investigating specific molecular markers related to the imbalance between cell death and proliferation, the capacity for tissue invasion and treatment sensitivity, could provide new insights for the identification of innovative treatments.

OSCC is a multifactorial disease, which is primarily associated with chronic tobacco, alcohol and betel nut use [3]. Genetic predisposition has also been implicated in oral tumorigenesis, and multiple genetic and epigenetic alternations are involved in the growth of cancer [4]. Aberrant DNA methylation plays a key role in carcinogenesis, leading to epigenetic silencing of tumor-suppressor genes involved in cell cycle regulation, apoptosis and DNA repair [5], [6]. DNA hypermethylation frequently occurs in pre-cancerous and cancer tissues [7]. In OSCC, p14, p16, MGMT, DAP-kinase and E-cadherin were frequently and extensively studies methylated genes [8]–[12]. DNA methylation is typically mediated by DNA methyltransferases (DNMT) and in mammalian genomes there are three DNMT genes; DNMT3a and DNMT3b are responsible for de novo methylation and modify unmethylated DNA, and DNMT1 is thought to be responsible for maintaining methylation patterns [13]–[15]. DNMT3b has been reported to participate in carcinogenesis of several cancer types including esophageal, gastric and lung cancer [16]–[18], but the role of DNMT3b in OSCC requires further investigation. We previously reported that activated IL-6 signaling was associated with the aggressive tumor behavior in oral cancer [19]. In oral cancer cells, IL-6 was reported to promote tumorigenesis by alterinig DNA methylation [20]. Accordingly, we examined the expression of DNMT3b in OSCC and its link with IL-6 signaling in the present study. The role of DNMT3b in OSCC *in vitro* and *in vivo*, and the correlation with the clinical outcomes of patients with OSCC using immunochemical staining analysis were also investigated.

## Materials and Methods

### Tissue specimens and characteristics of patients

The Institutional Review Board (IRB) of Chang Gung Memorial Hospital approved the present study (Permit Number: 98-3546B). The written consents were signed by the patients for their specimen and information to be stored in the hospital and used for research. The consent procedure was approved by IRB. Specimens were retrospectively collected from patients diagnosed with locally advanced OSCC who received curative treatment, and constructed into tissue microarrays (TMA) blocks for immunochemical analysis. In the study, TMA blocks were consisted of 125 OSCC cases with pathologic stage III–IV (buccal, n = 48; gingival, n = 24; lip, n = 10; tongue, n = 35; palate, n = 8) by AutoTiss 1000 (Ever BioTechnology, Canada). When blocks were available, hematoxylin and eosin-stained slides were re-evaluated by a pathologist to assess the quality of TMA slides. Data concerning the initial diagnosis, staging, pathological factors, recurrence and survival were collected (Table 1). Survival probability analyses were performed using the Kaplan-Meier method. Significance between group differences was assessed using the Sperman-rank test. Multivariate analyses were performed using a Cox regression model for overall survival. All statistical tests were two sided, with significance defined as p<0.05.

**Table 1:** Clinico-pathological characteristics of locally advanced oral cancer patients.

	No. of patients		
	IHC-DNMT3b (−)	IHC- DNMT3b (+)	*p* value
**patients**	49	76	
**Age**	37–77	31–84	
> = 55 y/0	25	40	0.86
<55 y/o	24	36	
**Differentiation**			
W-D	29	34	0.06
M-D	11	31	
P-D	5	8	
Unknown	4	3	
**Clinical stage**			
< = III	25	34	0.49
IV	24	42	
**Pathologic LN metastasis**			
N0	32	23	0.001*
N1–3	17	53	
**Risk factors**			
No	18	15	0.035*
Yes	31	61	
**Pathology stage**			
III	18	18	0.11
IV	31	58	
**Adjuvant RT**			
<4500 cGy	12	16	0.65
> = 4500 cGy	37	60	
**Failure**			
Local-regional failure and/or distant metastasis	6	40	0.000*
Disease free	43	36	

Risk factors including extracapsular spread, the presence of preineural, vascular or lymphatic invasion.

* *p* value<0.05.

### Immunohistochemical staining (IHC)

**Figure 1:**
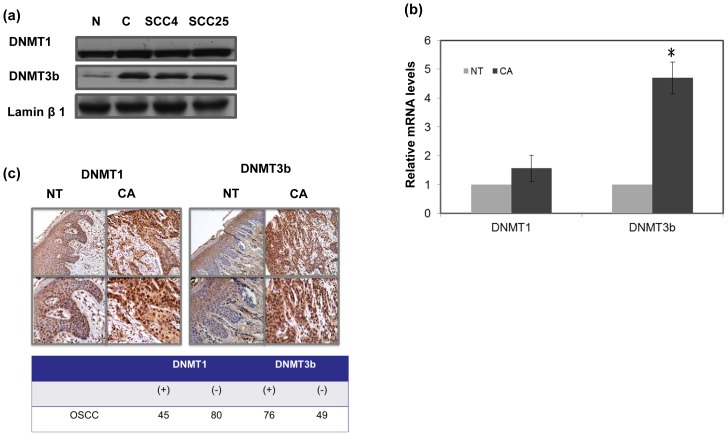
IHC staining for human oral cancer specimens by TMA slides. A. Levels of DNMT1 and DNMT3b were examined in cancer (CA) and non-malignant tissue (NT) by Western blotting analysis. B. Levels of DNMT1 and DNMT3b were examined in six specimens (paired cancer (CA) and adjacent non-malignant tissue (NT); by RT-PCR. The y-axis shows the ratio of target protein in cancer tissue divided by that in the non-malignant specimen. Columns, mean of three separate experiments; Bars, standard deviation (SD); *, P<0.05. C. Representative slides of IHC positive staining with DNMT1 and DNMT3b antibodies are shown by images using IPP (NT, adjacent non malignant epithelium; CA, oral cancer tissue). Images of representative slides are shown at magnifications of ×100 (upper row) and ×200 (lower row). * (+) means positive staining in cancer>adjacent NT.

Tissue sections from TMA blocks and formalin-fixed, paraffin-embedded tissues were cut into 4-µm sections, mounted on slides, deparaffinized with xylene and dehydrated using a graded ethanol series. Antigen retrieval, with the use of citric acid (pH 6.0) for 30 minutes, was followed by treatment with3% hydrogen peroxide. The slides were incubated overnight at 4°C with antibodies against specific proteins. Antibodies specific for p-H2AX, MMP-9, vascular endothelial growth factor (VEGF), and CD31 were obtained from Santa Cruz Biotechnology, Inc. (Santa Cruz, CA), Chemicon (Temecula, CA), Research & Diagnostics Systems, Inc. (Minneapolis, MN USA), and DNMT1 and DNMT3b were purchased from Abcam (Cambridge, MA), respectively. They were used at 1∶100 dilutions. After three washes in phosphate-buffered saline (PBS), the sections were incubated with biotinylated secondary antibody for10 min and stained with peroxidase-avidin and washed again in PBS; then 3-amino-9-ethylcarbazole solution was added. The sections were counterstained with hematoxylin. The IHC data were analyzed using Image Pro Plus 6.3 (IPP). Microvascular density (MVD) measurements were using CD31 staining Each immunoreactive endothelial cell cluster in contact with the selected field was counted. The data of IHC was examined by Good Speed scan slide scanning platform, and analyzed by Image Pro Plus 6.3 (IPP). This analysis demonstrated that the antibodies targeted proteins that were highly expressed in tumor epithelial cells (Fig. 1) compared with adjacent non-cancerous tissue. For histological evaluation of DNMT1 and DNMT3b staining, in addition to IPP analysis, slides were re-checked by a single pathologist. DNMT1 and DNMT3b immunoreactivity of a tissue sample was counted for the cells showed positive nuclear staining, regardless of cytoplasmic staining.The specimens were assessed using the semi-quantitative immunoreactive score (IRS). The IRS was calculated by multiplying the staining intensity (graded as: 0 = no, 1 = weak, 2 = moderate and 3 = strong staining) and the percentage of positively stained cells (0 = less than 10% of stained cells, 1 = 11–50% of stained cells, 2 = 51–80% of stained cells and 3 = more than 81% of stained cells). The criterion for positive staining is a specimen with an IRS scoring grade 2.

### Cell culture and reagents

The human oral cancer cell lines, SCC4 and SCC25, were obtained from Bioresource Collection and Research Center (BCRC). Human recombinant IL-6 antibody was obtained from R&D (Minneapolis, MN). The DNMT inhibitor 5-aza-2′-deoxycytidine was obtained from Sigma (St. Louis, MO). The DNMT3b-GFP silencing vector (psiRNA-h7SK vector expressing siRNA targeting human DNMT3B from the human 7SK RNA polymerase III promoter, and the GFP∶Zeo fusion gene which confers both reporter and antibiotic resistance activities) and GFP-control vector (Non-effective scrambled siRNA in GFP vector) were purchased from InvivoGen. Stable DNMT3b-silenced cancer cells were generated by transfecting SCC4 and SCC25 cells with the DNMT3b silencing vector and selected by culturing in medium containing Zeomycin for 4 weeks.

### Cell growth and clonogenic assay

The effects of DNMT3b on the cell growth rate were assessed using cells transfected with a DNMT3b silencing vector or control vector. To measure cell growth, 1×10^4^ cells per well were plated into 6-well dishes. At the indicated time-points, cells were trypsinized, collected and surviving cells counted using Trypan blue exclusion, from which the survival curves for SCC transfectants were established. In addition, clonogenic assay was used. Exponentially growing cells were incubated at 37°C for 10 days, and the plates were stained with crystal violet (Sigma) to aid colony counting. Colonies containing >50 cells were scored to determine plating efficiency and surviving fractions for each cell line.

### Immunoblotting

For western blotting of whole cells and oral tissue extracts, specimens were homogenized and/or treated with lysis buffer (Calbiochem, La Jolla, CA). The oral tissue specimens comprised six cancer tissue specimens and six non-malignant epithelium specimens. An NE-PER kit (Pierce, Rockford, IL) was used to separate nuclear and cytoplasmic proteins. To determine the *in vitro* effects of IL-6 Ab, and a DNMT inhibitor, proteins were extracted from cells in the presence or absence 5 µg/ml IL-6 neutralizing antibodies for 24 h or 5 µM 5-aza-2′-deoxycytidine for 36 h, respectively. The equal amount of protein was loaded in a 4–20% gradient SDS–PAGE gel, the protein was transferred onto nitrocellulose filters after separated in the gel. Blots were blocked in 2% BSA in TBST for 1 h, the membranes were incubated overnight (4°C) with antibodies against DNMT1, DNMT3b, VEGF, E-cadherin, STAT3, pSTAT3^Tyr705^, and MMP-9. The membranes were incubated with HRP conjugated secondary anti-goat, anti-mouse, or anti-rabbit antibodies (dilution 1∶1,000–1∶2,000), respectively, for 1 h at room temperature. Proteins were visualized by enhanced chemiluminescence. The membranes were reprobed with an antibody against r-tubulin or nuclear lamin to normalize protein loading.

### Real-time reverse transcription polymerase chain reaction (RT-PCR)

Real-time RT-PCR was performed on RNA extracted from cells and tissue specimens (six cancer tissue specimens and six non-malignant oral tissues; two specimens run in each lane). RNA (2 µg) was reverse-transcribed with a random primer to obtain the first cDNA strand. The sequences of primers for DNMT3b were 5′- GACTCGAAGACGCACAGCTG -3′/5′- CTCGGTCTTTGCCGTTGTTATAG′. To control for loading differences, a β-actin primer was used as a control. The optimized PCR was performed on an iCycler Iq multicolor real-time PCR detection system. Significant fluorescent PCR signals from carcinoma tissues were normalized to the mean value of the signals obtained from the non-malignant tissues and cells under control conditions.

### Immunofluorescence staining (IF)

Cells demonstrating exponential growth were seeded onto cover slips for immunofluorescence staining with or without treatment. At the indicated times after treatment, cells were fixed, permeabilized with 2% paraformaldehyde for 5 min and washed with PBST. The slides were incubated for 1 h at room temperature with antibodies against E-cadherin, DNMT3b, VEGF and MMP-9 for 1 h with a Texas Red-conjugated secondary antibody. The slides were counterstained with DAPI to visualize nuclei. After two washes with PBST, specific target proteins were visualized using a fluorescence microscope.

### Cell migration and cell invasion assay

Capacities for cell invasion were determined using a Cell Invasion Assay (Trevigen, Gaithersburg, MD). After incubation for 24 h, the number of cells in the bottom chamber was determined by measuring the fluorescent anion calcein, released from intracellular calcein acetoxymethylester. To validate experiments concerning cell migration, scratch assays were carried out. A 2 mm wide scratch was drawn across each cell layer using a pipette tip. The plates were photographed at the times indicated.

### Tumor xenograft model

This study was carried out in strict accordance with the recommendations in the Guide for the Care and Use of Laboratory Animals as promulgated by the Institutes of Laboratory Animal Resources, National Research Council, U.S.A. The protocol was approved by the Committee on the Ethics of Animal Experiments of Chang Gung Memorial Hospital (Permit Number: 2012080902). Eight-week-old female athymic nude mice were used as the xenograft tumor implantation model. In the ectopic tumor implantation model, cells (1×10^6^ tumor cells were injected subcutaneously per implantation, five animals per group) were implanted into the bilateral dorsal gluteal region. Tumor size was measured every three days after implantation (day 0). The tumor volume was calculated assuming an ellipsoid shape.

## Results

### Levels DNMT3b in oral cancer tissues

The expression of DNMT3b in the tissue specimens (malignant versus adjacent non-malignant) was examined by Western blotting, while mRNA was demonstrated by real-time RT-PCR (Fig. 1A–B). By the data, cancer demonstrated a significantly higher level of DNMT3b than non-malignant specimen. The IHC data of TMA slides confirmed this finding that DNMT3b was overexpressed in tumor tissues than the adjacent non-malignant epithelial tissues (Fig. 1C). Of the 125 oral cancer tissues assayed for DNMT3b and DNMT1 using IHC analysis, 76 (61%) gave DNMT3b positive immunoreactivity but only 36% (45/125) with DNMT1 positive staining. Furthermore, the role of DNMT3b in the clinical outcome of human OSCC was further evaluated using IHC staining. The patients were further classed as DNMT3b (+) or DNMT3b (−) on the basis of the IHC results. There was a positive correlation between the incidence of lymph node metastasis, disease recurrence and DNMT3b positive staining (Table 1). Eighty-three percent (40/46) of patients with disease recurrence (including local-regional failure and distant metastases) demonstrated positive staining for DNMT3b. In contrast, 46% (36/79) of patients classed as having disease-free status expressed DNMT3b. The findings suggest that DNMT3b staining might have the predictive value in the prognosis of oral cancer.

### Role of DNMT3b in tumor growth

**Figure 2:**
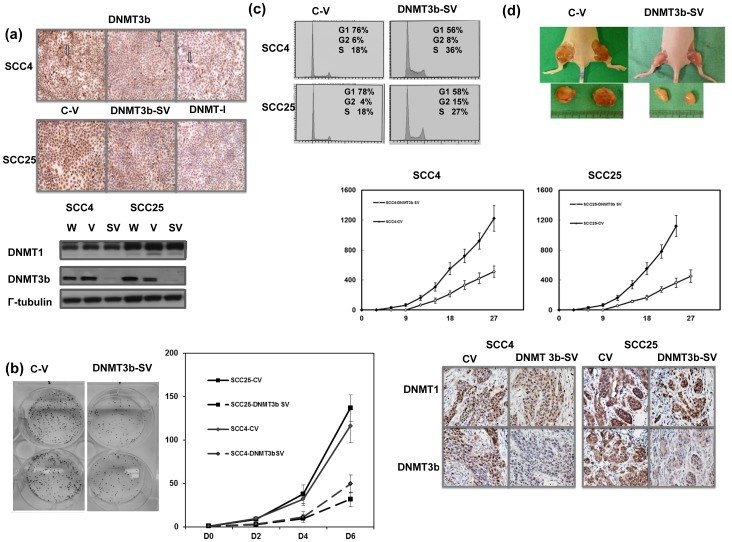
Role of DNMT3b in tumor growth *in vitro* and *in vivo*. A. Effects of DNMT3b silencing vector on the level of DNMT3b in SCC4 and SCC25 demonstrated by Western blotting and immunochemical staining *in vitro*. Images of representative slides are shown and the arrow indicates positive staining of DNMT3b in cells. (C-V, cells with control vector; DNMT3b-SV, cells stably transfected with DNMT3b silencing vector; DNMT-I, cells treated with DNMT inhibitor). B. Effect of DNMT3b on the proliferation of oral cancer cells as determined by viable cell counting and colony formation. The same number of cells (10^4^) were plated in each plate on day 0 and allowed to grow in their respective cultures. We counted the number of viable cells after incubation for 2, 4 and 6 days. The Y axis represents the viable cell number. Point, means of three separate experiments; bars, SD. *, *p*<0.05. C. Cells were analyzed by FACS for DNA content under control conditions or with DNMT3b inhibition. The data was demonstrated by represented pictures. D. Effects of DBMT3b silencing vector on the levels of DNMT1 and DNMT3b evaluated by IHC and xenograft tumor growth. Each point represents the mean of three separate experiments; bars, SD; *, *P*<0.05. Representative pictures are shown.

To investigate if alternating DNMT3b expression played a role in aggressive tumor growth, oral cancer cells were transfected with a DNMT3b-GFP silencing vector. As demonstrated in Figure 2A, the DNMT3b silencing vector significantly inhibited DNMT3b expression in cancer cells by Western blotting and immunochemical staining analysis. The effect of DNMT3b on tumor cell growth was determined by viable cell counting over six days and colony formation. The data by cellular experiments showed DNMT3b silencing vectors significantly inhibited tumor cell proliferation *in vitro* (Fig. 2B). The change in the cell cycle distribution was further measured. The DNMT3b silencing vector resulted in cell cycle arrest (Fig. 2C). As shown in Figure 2D by observation of xenograft tumors, inhibition of DNMT3b using silencing vectors significantly decreased tumor growth rate.

### Role of DNMT3b in tumor invasion

**Figure 3:**
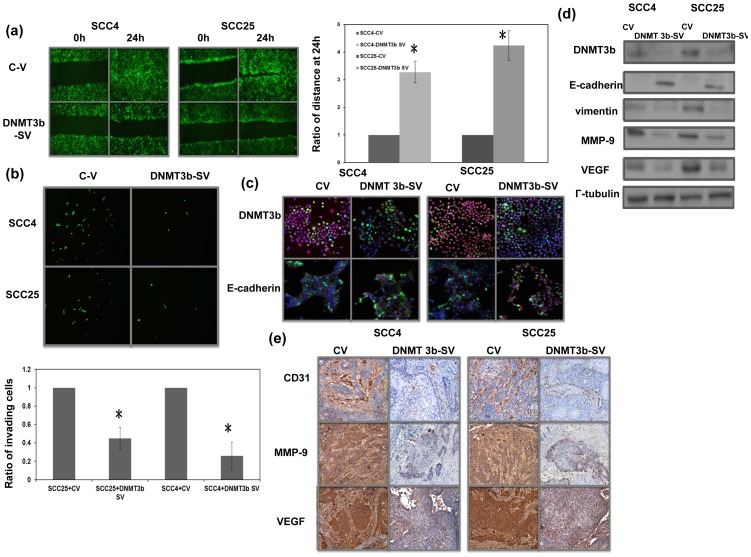
Role of DNMT3b in the tumor aggressiveness and EMT changes. A. The invasive capacity of oral cancer cells with or without the DNMT3b silencing vector was evaluated by migration scratch assays. The results from representative slides are shown. Column, mean of three separate experiments. Bars, SD. *, P<0.05. B. The invasive capacities of oral cancer cells with or without regulating DNMT3b were evaluated by invasion assay in SCC4 and SCC25 oral cancer cells. The results are shown by representative slides and quantitative data. *, *P*<0.05. C. The changes of E-cadherin in cells are evaluated and the results are shown by representative slides (upper row, immunofluorescent picture stained with Ab for DNMT3b and DAPI for nucleus; lower row, immunofluorescent picture stained with Ab for E-cadherin and DAPI for nucleus); (Blue: DAPI; Green: GFP-vector; Red: target protein). D. The changes in EMT associated proteins in cells with regulating DNMT3b were evaluated by Western blot analysis (C-V, cells with control vector; DNMT3b-SV, cells with DNMT3b silencing vector). E. IHC using MMP-9, CD31, and VEGF staining in formalin-fixed, paraffin-embedded tissues from xenograft tumors. DNMT3b silencing vectors were significantly reduced by the angiogenesis and EMT-related changes as compared with control vector.

There was a positive link between DNMT3b staining and LN metastasis and disease failure in patients with oral cancer (Table 1).As demonstrated using migration scratch and invasion assays, DNMT3b silencing vectors attenuated the invasion capacity of oral cancer cells *in vitro* (Fig. 3A–B). Epithelial mesenchymal transition (EMT) is a key event in invasiveness of a tumor [21]. Therefore, it was determined whether this was the mechanism underlying the effects of DNMT3b on oral cancer invasiveness. The DNMT3b silencing vector induced oral cancer cells to increase their epithelial characteristics as determined by changes in expression of E-cadherin, a hallmark of EMT [22] and vimentin (Fig. 3C–D). EMT is reported to be associated with a number of invasion-related factors including VEGF and MMP-9. Our data revealed that inhibition of DNMT3b by the silencing vector resulted in lower expression of VEGF and MMP-9. Angiogenesis is one of the mechanisms that promote tumor progression, and CD31-mediated endothelial cell-cell interactions are involved in angiogenesis [23]. Therefore, the vascular network within the tumor was measured by VEGF staining and microvascular density (MVD) analysis after CD31 staining. When oral cancer cells with control vectors and those with DNMT3b silencing vectors were subcutaneously implanted into mice, we found that the growth inhibiting effect induced by DNMT3b silencing vector associated with lower expression levels of EMT- and angiogenesis-related factor (Fig. 3E). Accordingly, we suggested that DNMT3b overexpression plays a role in tumor promotion, and the induction of angiogenesis and EMT might be the underling mechanisms.

### DNMT3b and IL-6 are linked with the clinical outcome of OSCC

**Figure 4:**
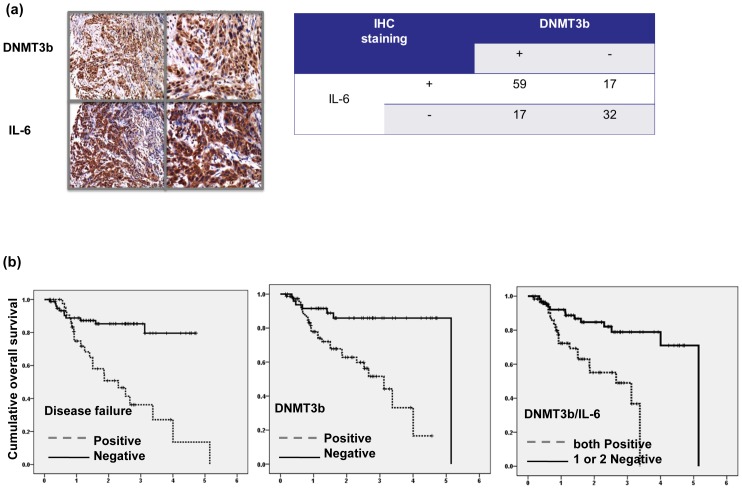
Level of DNMT3b and IL-6 in oral cancer clinical outcome. A. DNMT3b level was positively correlated with IL-6 expression in human oral cancer specimens (P<0.0001). Representative slides of a selected tumor specimen positively staining for both IL-6 and DNMT3b is shown at ×100 and 400 magnification. B. Survival differences according to developing disease failure or not, the positive staining of DNMT3b and positive staining for both DNMT3b and IL-6. The Kaplan- Meier overall survival curves shows that the positive groups were linked with shorter survival compared to that of respective negative group.

**Figure 5:**
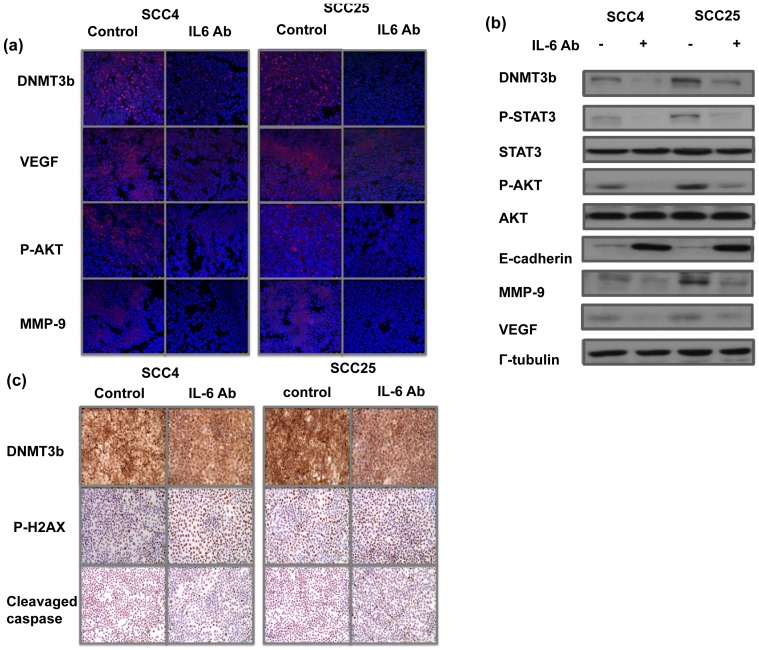
Role of IL-6 in the activation of DNMT3b. A. Effect of IL-6 on the levels of DNMT3b and EMT-related proteins are evaluated by immunofluoresence analysis *in vitro* and the results are shown by representative slides. B. Influence of blocking IL-6 on the level of DNMT3b, STAT3/AKT activation and EMT-related proteins are evaluated by Western blotting analysis. C. Effects of IL-6 on the level of DNMT3b and cell death proteins in SCC4 and SCC25 demonstrated by immunochemical staining *in vitro*.

Previously, we reported that activation of IL-6/STAT3 signaling induced aggressive tumor behavior and EMT changes in oral cancer [19]. Therefore, the correlation between DNMT3b and IL-6 in the clinical outcome of stage III–IV human OSCC was further estimated using IHC on TMAs consisting of 125 samples. Of the 125 tissue specimens assayed for DNMT3b and IL-6, both 59 (61%) were positive. As shown in Figure 4A, a significant positive correlation was observed in the cancer specimen that stained positively for DNMT3b and IL-6. Using univariate analysis, disease recurrence and positive staining for DNMT3b and IL-6 were all significantly linked with shorter survival (Table 2 and Figure 4B). Using multivariate analysis, expression of DNMT3b and disease recurrence was significant predictors for overall survival in the 125 oral cancer patients (Table 3). The findings highlight the contribution of DNMT3b staining to poor prognosis in oral cancer. By protein analysis, inhibition of IL-6 signaling resulted in decreased DNMT3b and EMT-related factors expression, associated with attenuated STAT3 and Akt activation (Fig. 5A–B). Moreover, Figure 5C demonstrated that decreased DNMT3b by IL-6 antibody increased DNA damage and cell death. Therefore, it is suggested that activation of IL-6 signaling might be responsible to the increased DNMT3b in oral cancers.

**Table 2:** Univariate analysis to determine factors associated with prognosis.

Variables	P value for Overall survival
Clinical stage	0.662
LN metastasis	0.245
Risk factor	0.74
Pathologic stage	0.74
Positive staining for DNMT3b	0.000*
Positive staining for IL-6	0.001*
Positive staining for DNMT3b and IL-6	0.000*
Disease failure	0.000*

Risk factors including extracapsular spread, the presence of preineural, vascular or lymphatic invasion.

* *p* value<0.05.

**Table 3:** Multivariate analysis to determine molecular markers associated with prognosis (OS) of patients.

Variables	Odd ratios	95% confidence interval	p
DNMT3b staining	3.712	1.178–11.699	0.025*
Disease failure	2.523	1.102–5.777	0.029*
Pathologic stage	1.224	0.463–3.238	0.684
LN metastasis	0.942	0.426–2.086	0.883
Risk factor	0.597	0.211–1.694	0.333

* *p* value<0.05.

## Discussion

It has been reported that a switch to accumulating DNA hypermethylation could be caused by over-expression of DNA methyltransferases [15]. Epigenetic gene silencing, promoted by DNMTs, has been observed in various malignancies, supporting the claim that DNMT genes are over-expressed in human cancers and during cellular transformation [24]–[26]. In the present study, IHC of TMA slides revealed that the level of DNMT3b expression was higher in 76 (61%) cancer specimens than in adjacent non-malignant oral epithelium. We further evaluated if DNMT1 was overexpressed in OSCC using IHC of TMA slides. The data revealed that only 45 (36%) cancer specimen had higher DNMT1 compared to adjacent non-malignant epithelial tissues. Identification, development and selection of molecular targets are important in cancer therapy. The predictive powers of DNMT3b were further examined in terms of the clinical outcome of oral cancer. Using univariate analysis, enhanced expressions of DNMT3b were significantly associated with a higher incidence of lymph node metastasis, a higher recurrence rate after treatment and shorter survival. Using multivariate analysis, disease failure and enhanced expression of DNMT3b were significantly associated with shorter overall survival. To further investigate whether DNMT3b was responsible for aggressive tumor growth of OSCC, we suppressed DNMT3b in oral cancer cells using a silencing vector. Data obtained from cellular experiments and xenograft tumor growth experiments revealed that inhibition of DNMT3b resulted in slower tumor growth. Additionally, inhibition of DNMT3b was associated with attenuated invasiveness. The molecular and phenotypic changes involved in the transformation of an epithelial cell to a mesenchymal cell type appear to be functionally relevant to the invasive characteristics of epithelial tumors [21]. It has been reported that EMT is associated with OSCC progression [27], [28]. At the molecular marker level, EMT is characterized by a loss of E-cadherin and increased expression of vimentin and invasion-related factors. The DNMT3b silencing vectors induced an increase in E-cadherin and decreases in VEGF and MMP-9 in oral cancer cells. Additionally, angiogenesis is one of the mechanisms that promote tumor progression, and CD31 mediated endothelial cell-cell interactions involved in angiogenesis. By our data, the inhibition of DNMT3b attenuated tumor growth, associated with decreased CD31 and VEGF expressions in tumors. These findings indicated that DNMT3b might mediate the aggressive tumor growth in oral cancer, and the promotion of EMT and angiogenesis is one of the underlying mechanisms.

**Figure 6:**
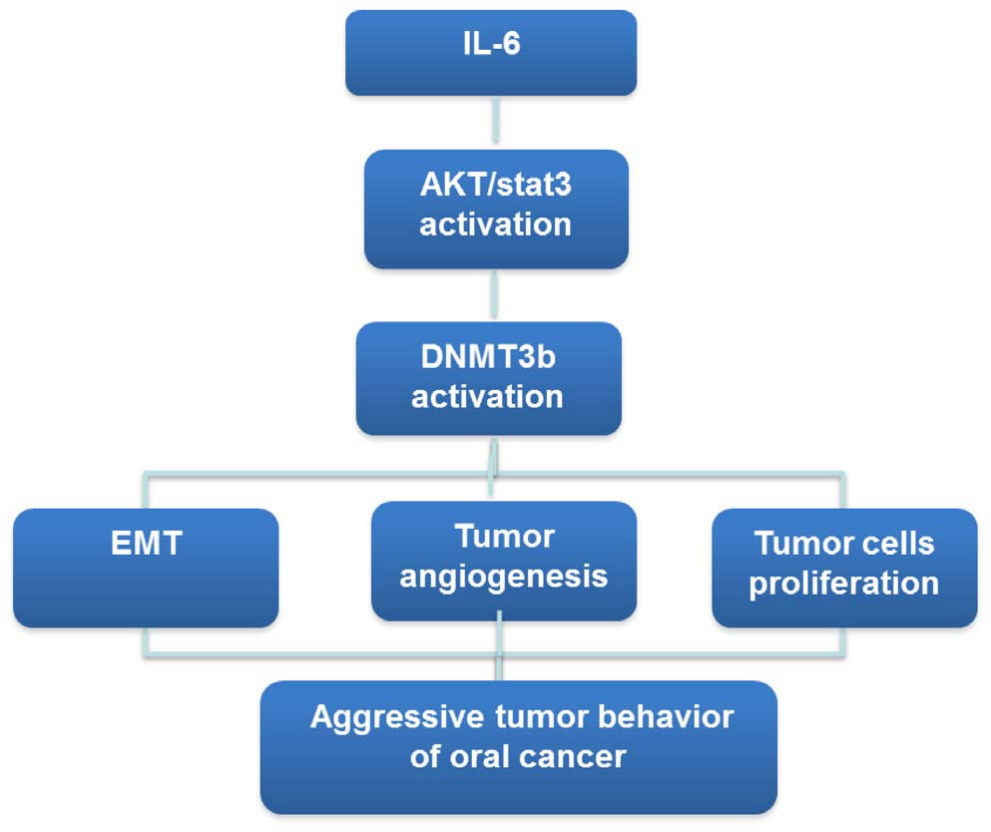
IL-6-DNMT3b signaling pathway in oral cancer.

OSCC development is in part facilitated by chronic epithelial irritations and this tumor is more frequent in smoker or who consume excessive amount alcohol. IL-6, is a proinflammatory cytokine that mediates chronic inflammation and has been reported to play an important role in inflammation-driven oral carcinogenesis [29], [30]. We previously reported that activated IL-6 pathways could be responsible for more aggressive tumor growth noted in oral cancer, and high activated STAT3 and p-AKT level was induced by IL-6 and linking IL-6 to tumorigenesis [19]. It is reported that IL-6 levels are elevated in this neoplasm and IL-6 is considered a bad prognostic factor in oral cancer. IL-6 secretion in oral squamous cancer is facilitated by the microenvironment, in particular by stromal derived factor-1 [29]. Moreover, numerous projects have demonstrated that IL-6- induced hypermethylation and gene silencing may be mediated by DNMTs [18], [20], [31]–[33]. Taken together, we proposed that chronic inflammation stress in the oral cavity may promote tumorigenesis mediated by increased IL-6 to induce aberrant DNA methylation via increased DNMT3b activity. In the present study, IHC data obtained from clinical samples demonstrated that DNMT3b expression was significantly correlated with IL-6 staining. The tumor aggressiveness noted in IL-6 positive OSCC may be in part via the overexpression of DNMT3b. To test the hypothesis, the relationship between IL-6 signaling and DNMT3b in OSCC was further examined in the present study to see whether regulation of IL-6 signaling results in changes of DNMT3b expression. By the experiments in which IL-6 signaling was suppressed by IL-6 antibody, we found that activated IL-6 signaling plays an important role in DNMT3b activation associated with activated STAT3 and PI3K in oral cancer cells. The data obtained from the present study revealed that the increased DNMT3b induced by IL-6, which might be mediated by the activation of STAT3 and PI3K signaling, is critical in tumor aggressiveness and prognosis of oral cancer. We therefore outlined the main signaling pathways that are thought to link IL-6 and DNMT3b to oral cancer (Fig. 6).

In conclusion, inflammation and methylation are both believed to play key roles in oral tumorigenesis, however, inflammation-driven effects on methylation need further investigation in this disease. This project established IL-6-DNMT3b axis is likely to be important in the prognosis of OSCC. Identification, development and selection of molecular targets are important in cancer therapy. The data support the emerging hypothesis that the IL-6-DNMT3b axis is a significant predictor, and targeting DNMT3b may represent a promising treatment strategy for OSCC.
